# *Plasmodium falciparum* Transfected with Ultra Bright NanoLuc Luciferase Offers High Sensitivity Detection for the Screening of Growth and Cellular Trafficking Inhibitors

**DOI:** 10.1371/journal.pone.0112571

**Published:** 2014-11-13

**Authors:** Mauro F. Azevedo, Catherine Q. Nie, Brendan Elsworth, Sarah C. Charnaud, Paul R. Sanders, Brendan S. Crabb, Paul R. Gilson

**Affiliations:** 1 Macfarlane Burnet Institute of Medical Research and Public Health, Melbourne, Victoria, Australia; 2 Monash University, Melbourne, Australia; 3 University of Melbourne, Melbourne, Australia; Bernhard Nocht Institute for Tropical Medicine, Germany

## Abstract

Drug discovery is a key part of malaria control and eradication strategies, and could benefit from sensitive and affordable assays to quantify parasite growth and to help identify the targets of potential anti-malarial compounds. Bioluminescence, achieved through expression of exogenous luciferases, is a powerful tool that has been applied in studies of several aspects of parasite biology and high throughput growth assays. We have expressed the new reporter NanoLuc (Nluc) luciferase in *Plasmodium falciparum* and showed it is at least 100 times brighter than the commonly used firefly luciferase. Nluc brightness was explored as a means to achieve a growth assay with higher sensitivity and lower cost. In addition we attempted to develop other screening assays that may help interrogate libraries of inhibitory compounds for their mechanism of action. To this end parasites were engineered to express Nluc in the cytoplasm, the parasitophorous vacuole that surrounds the intraerythrocytic parasite or exported to the red blood cell cytosol. As proof-of-concept, these parasites were used to develop functional screening assays for quantifying the effects of Brefeldin A, an inhibitor of protein secretion, and Furosemide, an inhibitor of new permeation pathways used by parasites to acquire plasma nutrients.

## Introduction

Malaria is a life-threatening disease caused by five species of parasites belonging to the genus *Plasmodium*, and includes *P. falciparum*, *P. vivax*, *P. ovale, P. malariae* and *P. knowlesi*. Despite successful efforts to reduce mortality through transmission control and patient treatment, there are still over 200 million clinical cases per year and tragically about 600,000 deaths [1]. The appearance and spread of multidrug resistant strains of the most deadly species, *P. falciparum* threatens to revert the mortality and morbidity reductions achieved so far and future control and eradication strategies must include the discovery of new anti-malarial compounds.

Recently, extensive high throughput screens of proprietary and publicly available libraries have identified thousands of potent compounds that inhibit the growth of *P. falciparum* blood stages in *in vitro* culture [2]–[5]. There is now a pressing need to identify the targets of these compounds and the aspects of parasite biology that are inhibited by them. Although the structural family to which some compounds belong may indicate their likely targets, for most compounds the targets are completely unknown. This is further exacerbated by the fact that even if the targets could be identified by complex and costly laboratory investigation, the functions of more than half of the parasite’s proteins are still unknown. This therefore makes it very difficult to deduce what a compound’s mechanism of action might be, making it problematic to evaluate if the compound is worth further development.

In the bloodstream, red blood cells (RBCs) are infected by the short-lived extracellular merozoite form of the malaria parasite. During invasion of the RBC, the malaria parasite envelops itself in the parasitophorous vacuole membrane (PVM). In order to grow rapidly and replicate, *Plasmodium* parasites extensively modify the RBC host by exporting hundreds of proteins across the PVM into the RBC cytosol and some then further out to the RBC plasma membrane [6]–[8]. While the function of most of these exported proteins is unknown, those that have been investigated appear to be associated with virulence related functions such as cytoadherence to vascular endothelium and strengthening of the host cell cytoskeleton [9], [10]. Parasites also cause their host erythrocytes to become more porous so blood plasma nutrients can be acquired for rapid growth. At least one secreted parasite protein called RhopH1, has been shown to help establish these new permeation pathways (NPPs) but it is not clear if other parasite proteins, potentially exported into the host, also play a role [11]. Protein export is a unique aspect of the parasite’s biology and is essential for their survival and contributes greatly to disease pathology. Recently a novel protein export machine called PTEX, has been identified that resides in the PVM [12] and appears to selectively transport proteins across the membrane into the RBC cytoplasm [13], [14]. PTEX appears to constitute a single portal through which hundreds of exported proteins must pass. Therefore, inhibition of PTEX or of any complex required for protein export is likely to be highly effective against the parasite, as this will simultaneously disrupt the functions of 100 s of essential or virulence related exported proteins given they will not be able to reach their functional destinations.

So far, assays used to screen molecules active against *P. falciparum* parasites have mostly been based on measuring inhibition of overall growth of asexual blood stages, and recently of sexual stages [15], [16]. Growth inhibition assays have the advantage of being able to test in a high throughput manner large chemical libraries to identify potential hits, but have the disadvantage of not allowing biological validation of the actual targets of candidate drugs. Conversely, specific assays such as the detection of protein export in *P. falciparum* have been established by expressing fluorescent reporters such as the green fluorescent protein (GFP) fused at its N-terminal with a previously characterized protein export element (PEXEL) [17]. To use such approaches to screen for export inhibitors would be slow and laborious requiring sophisticated microscopes and highly trained personnel.

Luciferase (Luc) is the enzyme required for bioluminescence in a variety of organisms, and it has been adapted as a reporter in several cell types. The firefly and Renilla luciferases, from *Photinus pyralis* and *Renilla reniformes* respectively, have been expressed in *Plasmodium spp.* without any apparent toxicity [18],[19]. This reporter has been used to study several aspects of parasite biology [20]–[22] and for growth/drug inhibition assays [4], [16], [23]–[27]. In addition, being an exogenous protein allows Luc to be expressed in fusion with other proteins, including trafficking signal motifs. Protein trafficking in *P. falciparum* has been investigated using Luc, but the inefficient fractionation technology available impaired conclusive results regarding protein export [28].

Here NanoLuc (Nluc), a new smaller and brighter luciferase from deep-sea shrimp [29], is evaluated as a reporter in *P. falciparum*. Parasites engineered to express cytosolic, secreted and exported forms of Nluc have been developed, and new affordable and simple assays to quantify protein secretion and export, NPP functions as well as parasite growth are described.

## Materials and Methods

### Plasmid Construction

Nluc coding sequence was retrieved from pNL1.1 (Promega) digested with *Nco*I and *Xba*I and cloned in the same restriction sites of pPf86 [19] to generate pPfNluc. The stable expression vector pEF-Nluc was made cloning Nluc in the *Nco*I and *Spe*I sites of pEF-Luc [30]. To express a version of Nluc that could be exported into the erythrocyte compartment, a synthetic gene (Genscript) encoding the first 113 amino acids of the *P. falciparum* exported protein hyp1 (PF3D7_0113300), was cloned in pEF-Nluc via *Xho*I and *Nco*I sites, generating pEF-PEXEL-Nluc. The secreted Nluc was made cloning a synthetic gene encoding the first 23 amino acids of *P. falciparum* MSP1 (PF3D7_0930300) in *Xho*I and *Nco*I sites of pEF-Nluc, generating pEF-SP-Nluc.

### Parasite Culturing and Transfection

*P. falciparum* 3D7 parasites were cultured as per [31] in RPMI-HEPES media supplemented with L-glutamine (Sigma) and Albumax II (Invitrogen). Trophozoite stage parasites were transfected by feeding with erythrocytes electroporated with 100 µg of plasmid DNA [32], [33]. For transient transfections, parasites were harvested 4 days later. For stable transfections, parasites were cultured on 2.5 nM WR99210 until a transfected population was established.

### Luciferase Assay

Unless otherwise stated all bioluminescence reagents are from Promega. Transiently transfected parasites were lysed with 0.1% saponin and washed twice in PBS to remove the hemoglobin. For firefly luciferase, parasite pellets were re-suspended in 20 µL of 1x Luciferase Cell Culture Lysis Reagent, mixed with 100 µL of Luciferase Assay Reagent, containing the Luciferase Assay Substrate Luciferin previously dissolved in Luciferase Assay Buffer, and immediately measured for 30 seconds in a FLUOstar Omega Luminometer (BMG Labtech) with the gain adjusted to maximum. For Nluc, parasite pellets were re-suspended in 100 µL of PBS, mixed with 100 µL of 2x Nano-Glo Luciferase Assay Reagent and measured in the luminometer with the same gain and for the same time used for firefly luciferase. Nano-Glo Luciferase Assay Reagent was made by adding one volume of Nano-Glo Luciferase Assay Substrate to 50 volumes of Nano-Glo Luciferase Assay Buffer as recommended by the manufacturer. Cultures of stably transfected Nluc parasites, were diluted in RPMI to 0.1% hematocrit, mixed with 1 volume of Nano-Glo Luciferase Assay Reagent and measured for 2–10 seconds as indicated in each experiment in a luminometer with the gain reduced by 10% for the sample with the highest signal on the plate to prevent saturation.

### Chloroquine Sensitivity

Nluc ring stage parasites were incubated with chloroquine (CQ) concentrations varying from 52 pM to 4 mΜ in 5-fold increments. After 3 days, cultures were frozen and stored at −80°C. Frozen cultures were thawed immediately before Nluc or lactate dehydrogenase (LDH) quantification [34]. Three experiments were done in duplicate with Nluc independently transfected lines. IC_50_ was determined by non-linear regression analyzed by GraphPad Prism software.

For transient transfections, parasites at trophozoite stage were incubated with 400 µL of erythrocytes previously electroporated with 150 µg of pPfNluc and left to invade for one day in a culture volume of 10 mL (4% hematocrit). Culture volume was increased to 40 mL (1% hematocrit), cultures split in 5 mL volumes and CQ added at each concentration. Cultures were maintained for 3 days and Nluc quantified after hemoglobin removal by saponin lysis as described in the Luciferase Assay section. Results correspond to 3 independent experiments. For each assay 100% growth corresponds to parasites grown in absence of CQ and 0% growth to parasites grown in supra lethal dose of the drug (4 mM). The robustness of the assays were evaluated by calculating the statistical parameters Z’ value, signal to background ratio (S/B), signal to noise ratio (S/N), %CVmax and %CVmin as previously described [35]. The Z’ value was calculated according to the formula: Z’ = 1–(3 σ_c(+)_+3 σ_c(−)_)/|µ_c(+)_−µ_c(−)_|, where µ_c(+)_ and σ_c(+)_ correspond the mean and standard deviation of the positive control (100% growth) and µ_c(−)_ and σ_c(−)_ correspond the mean and standard deviation of the negative control (0% growth) respectively. The S/B was calculated as µ_c(+)_/µ_c(−)_ and S/N as (µ_c(+)_−µ_c(−)_)/σ_c(−)_. CVmax and CVmin were calculated as 100×(σ_c(+)_/µ_c(+)_) and 100×(σ_c(−)_/µ_c(−)_) respectively.

### Immunofluorescence Microscopy

Nluc, SP-Nluc or PEXEL-Nluc parasites were settled on poly-L lysine coated wells of a Lab-Tek chamber slide and fixed in 4% paraformaldehyde and 0.0075% gluteraldehyde in PBS [36]. Parasites were probed with an anti-EXP2 monoclonal [37] and rabbit anti-NanoLuc IgG (a kind gift from Lance Encell, Promega) all at 20 µg/mL. Images were acquired with a Zeiss AxioObserver inverted microscope and processed with ImageJ.

### Sub-Cellular Fractionation

Equinatoxin II was expressed in *E. coli* and purified as previously described [38] and stock aliquots at [1 mg/mL] were kept at −80**°**C, thawed and diluted 10-fold in PBS just prior to use. 50 µL of total parasite culture at 1% hematocrit was transferred to 1.5 mL tube and washed with 0.5 mL of RPMI to remove Nluc from the media. After centrifuging at 3,000 g for 3 min, 0.5 mL of the supernatant was discarded. To the 50 µL of culture left in the tube, 1 volume of equinatoxin in PBS was added. After gently mixing for 5 min, 400 µL of RPMI was added and the samples centrifuged at 6,000 g for 3 min. 50 µL of the supernatants were recovered and transferred to a 96 well plate, corresponding to the RBC fraction. Most of the remaining supernatant was then removed and discarded leaving about 30 µL in the tube. This represents 6% contamination, necessary to avoid accidental removal of the cells on the bottom of the tube. 470 µL of 0.01% saponin diluted in RPMI was added, and gently mixed for 2 min. The sample was centrifuged at 6,000 g for 3 min and 50 µL of the supernatant transferred to a 96 well plate, which corresponds to the PV fraction. Most of the solution was discarded, leaving about 30 µL in the tube. 470 µL of RPMI was added to dilute the sample, which was gently mixed and 50 µL transferred to a 96 well plate to become the parasite fraction. Just prior to measuring in the luminometer, 50 µL of Nano-Glo Luciferase Assay Reagent was added to each sample.

### Brefeldin A (BFA) incubation

Trophozoite stage cultures were incubated for 6 hours with 5 mg/mL BFA (Sigma) and harvested for RBC fractionation and Nluc quantification. DMSO was used to resuspend BFA and served as a negative control.

### New Permeation Pathway assay

Parasite culture expressing PEXEL-Nluc were washed to remove excess Nluc from the culture media. The culture containing 1–5% trophozoite stage parasites was diluted to 1% hematocrit. The culture was incubated with 5-fold the final concentration of furosemide for 20 min at RT. 10 µl of culture was then added in triplicate to new wells of an opaque 96-well plate (Nunc). 40 µl of sorbitol lysis buffer (280 mM sorbitol, 20 mM Na-HEPES, 0.1 mg/ml BSA, pH 7.4) or PBS was then added. The cells were then pelleted and the supernatant was collected at each time point and frozen. The supernatant was then mixed with 2x Nano-Glo assay reagent buffer and luminescence was measured. Alternatively, a 1/2000 dilution of Nano-Glo was added directly to the sorbitol lysis buffer or PBS and luminescence was measured at each time point.

The statistical parameters Z’ value, S/B, S/N, %CVmax and %CVmin were calculated in order to determine the robustness of the assay at each time point where reporter activity was measured. Parasites incubated with 0 µM or 25 µM furosemide served as positive and negative (background) controls respectively. Experiments were performed in triplicates and repeated once.

## Results

### NanoLuc expression in *Plasmodium falciparum*

**Figure 1:**
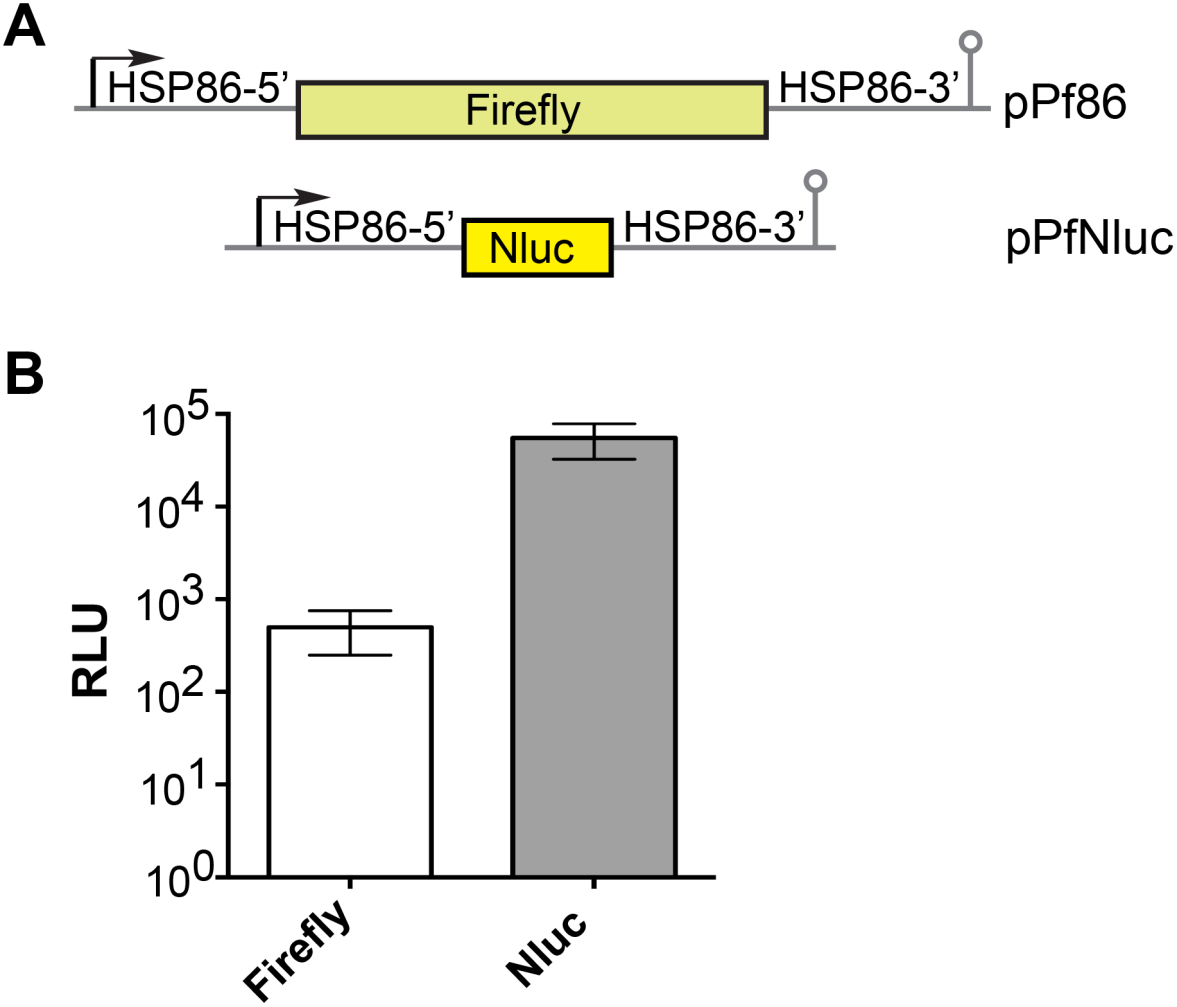
NanoLuc (Nluc) luminescence in *Plasmodium falciparum.* (A) Diagrams of firefly and Nluc reporter vectors. (B) pPf86 and pPfNluc were transfected in trophozoite stage parasites and luciferase activity in relative light units (RLU) determined 4 days later. Note that the RLU of each luciferase was measured in its own optimal substrate ie, Nluc with Nano-Glo and Firefly with D-luciferin. Mock-transfected parasites were used as negative control and to determine background luminescence, which was then subtracted from firefly and Nluc activities. The result represents the mean of 3 independent transfections ± standard deviation.

Nluc is an ATP independent luciferase nearly two orders of magnitude brighter than firefly and *Renilla* luciferases when expressed in mammalian cells [29]. To evaluate if Nluc would perform similarly in *P. falciparum*, the reporter plasmid pPfNluc was made by replacing the firefly luciferase gene in pPf86 [19] with that encoding Nluc (Fig. 1A). With both firefly luciferase and Nluc under control of the same Hsp86 promoter, the plasmids were electroporated into uninfected RBCs and then fed to *P. falciparum* trophozoite stage cultures so that the parasites would take up the plasmid when they invaded the electroporated RBCs. Four days later, the so-called transient transfectants were assayed for luciferase activity after saponin lysis which selectively permeabilises the RBC to facilitate the removal of haemoglobin, a powerful quencher of emitted light. Extracts of PfNluc parasites when provided with its optimised furimazine substrate (Nano-Glo Luciferase Assay Reagent) produced a strong signal (Fig. 1B), but extracts from the pPf86 parasites did not (data not shown). Firefly luciferase activity in the pPf86 transfectants could only be detected when provided with its D-luciferin substrate diluted in the Luciferase Assay Buffer (Fig. 1B). Extracts of mock-transfected parasites were used as negative controls and to determine the background luminescence for each substrate. After subtracting the background activities, Nluc was approximately 117-fold brighter then firefly luciferase, demonstrating Nluc can be expressed in *P. falciparum* and similar to mammalian cells is considerably brighter than firefly luciferase (Fig. 1B).

### Stable expression in *P. falciparum*

**Figure 2:**
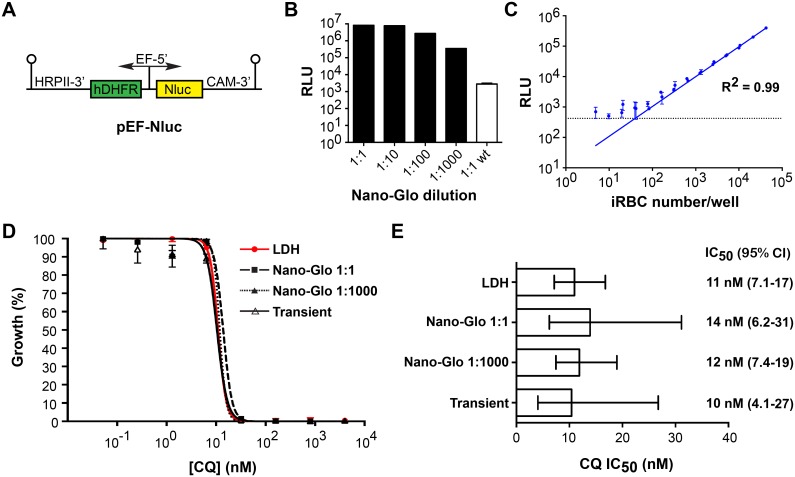
*Plasmodium falciparum* stably expressing Nluc. (A) The Nluc gene was cloned in the pEF vector for stable expression in *P. falciparum*. (B) Aliquots (100 µL) of cultures at 1% hematocrit and 5% parasitemia corresponding to 500,000 *P. falciparum* infected RBCs transfected with pEF-Nluc were mixed with 1 volume of Nano-Glo Luciferase Assay Reagent and reporter activity measured (1∶1). Nano-Glo Luciferase Assay Reagent was further diluted in 10-fold increments in Luciferase Cell Culture Lysis Reagent and used to determine reporter activity of the same culture. As a negative control, wild type parasites (wt) were mixed 1∶1 with Nano-Glo Luciferase Assay Reagent. (C) Parasites stably transfected with pEF-Nluc were diluted in 2-fold increments in RPMI + RBC maintaining 0.5% hematocrit. For each sample, 10 µL of the culture dilutions were mixed with 40 µL of Nano-Glo diluted 1∶400 in water and measured in the luminometer for 2 s with the gain adjusted 10% below saturation for the brightest sample. The solid line represents the mean RLU after linear regression. The dashed line represents the background +3 standard deviations. (D) Nluc expressing parasites were cultured in varying concentrations of chloroquine (CQ) and their growth determined by the LDH standard method or by measuring reporter activity using Nano-Glo Luciferase Assay Reagent at its standard dilution (1∶1) or diluted 1∶1000 as described in (C). Similarly, wild type parasites were transiently transfected with pPfNluc and their growth determined using Nano-Glo Luciferase Assay Reagent (Transient). IC_50_ was calculated by non-linear regression and represents the mean of 3 experiments. (E) The IC_50_s determined in D were plotted with 95% confidence intervals (CI).

*P. falciparum* tolerance for Nluc expression in continuous culturing was next investigated. The stable transfection vector pEF-Nluc was made by replacing the firefly luciferase of pEF-Luc [30] with Nluc (Fig. 2A) and this vector was stably transfected in *P. falciparum*. To confirm that the transfected parasites were expressing Nluc, 100 µL of parasite culture at 1% hematocrit and 5% parasitemia, corresponding to 500,000 infected red blood cells (iRBC), were washed and resuspended in the original volume of PBS. The cells were then lysed in 100 µL of Nano-Glo Luciferase Assay Reagent (referred to as 1∶1 in Fig. 2B) and this, measured for 10 seconds in the luminometer with its gain adjusted to 10% below saturation, yielded almost 10^7^ RLU, which is the value where the signal saturates. The high signal strength prompted us to determine if the Nano-Glo Luciferase Assay Reagent could be more economically used and so it was diluted in 10-fold increments in Luciferase Cell Culture Lysis Reagent, part of the Luciferase Assay System (Promega), and tested with the same number of Nluc expressing parasites in the same conditions. A 10-fold dilution of Nano-Glo decreased the signal by less than 10%, suggesting the substrate was in excess for the amount of enzyme present in the extract (1∶10, Fig. 2B). The 100 and 1,000-fold dilutions resulted in signal decreasing about 3 and 24-fold, respectively (1∶100 and 1∶1000, Fig. 2B). In spite of the lower signal detected, 1,000-fold dilution of Nano-Glo still permitted detection at a S/B ratio of approximately 125 (1∶1000 versus 1∶1 *wt*, Fig. 2B).

High throughput applications require the reporter detection to correlate linearly with cell number and the reagent to be stable for the duration of the experiment. We determined that 1∶1000 dilution is stable for up to 20 minutes (data not shown) and so that this dilution was only used for assays that lasted less than that. A slightly lower dilution (1∶500) was stable for up to 40 minutes (data not shown) and this was chosen for most of the assays. In order to determine if detection of reporter activity using the 1∶500 dilution would correlates linearly with parasite number, Nluc activity was measured in samples containing a range of 5–42,500 iRBC at the same hematocrit (Fig. 2C). The luminometer’s gain was set to 10% below saturation for the brightest sample and measurements performed for 2 s. Linear correlation of RLU and parasite number could be achieved from 78–42,500 iRBC, corresponding to a parasitemia of 0.015–8.5% for that specific experiment (Figure S1). Setting the gain to maximum did not improve the detection threshold (Figure S2A), but saturated the signal at about 10,000 iRBC. Increasing both the gain to max, the measurement time to 10 s, and using the standard Nano-Glo dilution (1∶1) allowed detection in the linear range down to approximately 10 iRBC, but the signal saturated at just over 600 iRBC, providing a very narrow window of detection in the linear range (Figure S2B). In order to assure detection in the linear range, all subsequent experiments contained <50,000 iRBC, with the gain set to 10% below saturation for the brightest sample and thus the absolute RLU values cannot be compared among different experiments. Using another cell line with exported Nluc (described next), detection in the linear range was achieved in the range of 71–73,500 iRBC (Figure S3).

The possibility of working at such a high Nano-Glo dilution and keeping a high S/B ratio raised the question whether Nluc could be used as an inexpensive high throughput assay for quantification of *P. falciparum* growth in the presence of inhibitors. To test this, the Nluc parasites, which were transfected forms of the chloroquine-sensitive 3D7 strain, were grown for 3 days in the presence of varying concentrations of chloroquine (CQ). Initial parasitemias were 0.5–1% ring stage parasites, at 1% heamatocrit in a total culture volume of 100 µL. After three days the parasitemias were about 2.5–5% of late trophozoite/schizont stage parasites and the cells present in 10 µL of the culture were harvested and reporter activity detected using the Nano-Glo Luciferase Assay Reagent in its standard dilution or diluted 1,000-fold as in Figure 2B. As a control, the remainder of the cultures were used to quantify growth by the benchmark lactate dehydrogenase (LDH) assay where the activity of endogenous LDH enzyme was measured [34]. The growth curves and IC_50_ were similar for all the methods applied, suggesting Nluc performs similarly to LDH and that 1,000-fold Nano-Glo Luciferase Assay Reagent dilution can be used (Fig. 2D–E).

A caveat of using luciferase to quantify cell growth is that the parasites have to be already stably transfected, which can take several weeks for *P. falciparum*. The RLU levels detected in the transient transfections with pPfNluc (Fig. 1B) suggested the signal might be strong enough to be used for a growth assay. To test that, erythrocytes electroporated with pPfNluc were fed to wild type parasites at trophozoite stage and left to invade for 1 day. After that, varying concentrations of CQ were added and parasites cultured for 3 more days when Nluc and LDH activities were determined. Both the growth curve (Fig. 2D) and the IC_50_ were similar to what had been determined for the stable transfected parasites measured by Nluc or by LDH activities (Fig. 2E).

Statistical parameters were calculated to evaluate the robustness of the assay and whether it could be suitable for high throughput drug screening (Table 1). Z’ values for CQ sensitivity assay using the stable transfected line and the standard or 1∶1000 Nano-Glo dilutions were >0.9 and similar to the LDH assay. The assay using transiently transfected parasites produced a significantly lower, but still suitable (>0.6) Z’ value, which was caused mainly by a lower S/B ration and higher variation of both positive and negative controls.

**Table 1:** Summary of the assay parameters from the growth assays.

	Assay and reagent dilution			
	Nano-Glo 1∶1	Nano-Glo 1∶1000	Transient^1^	LDH
**Z’-value**	0.96±0.02	0.95±0.02	0.69±0.11	0.93±0.03
**S/B ratio**	114±52	70±26	21±13	5±0.7
**S/N ratio**	1397±84	687±221	182±48	163±75
**%CV_max_**	1.36±0.60	1.56±0.47	9.20±3.99	1.23±0.97
**%CV_min_**	8.01±3.28	9.92±0.66	10.50±4.59	2.84±1.31

1 refers to luminescence measured from transiently transfected parasites using the standard Nano-Glo dilution.

### Targeting Nluc beyond the parasite membrane

**Figure 3:**
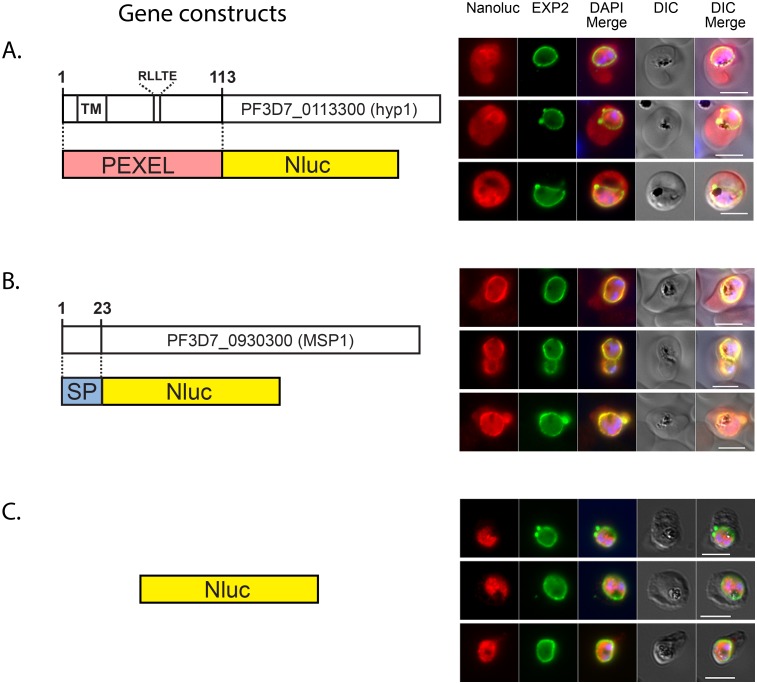
NanoLuc is targeted to the PV and to the RBC. Diagrams of gene constructs and the IFA images are shown on the left and right respectively. Nluc fused at its N-terminus to (A) the N-terminal region of an exported protein (PEXEL), (B) to a secretion signal peptide (SP) or (C) original (cytosolic). The gene encoding each fusion protein was cloned in the pEF vector. Transfected parasites were analysed by IFA using antibodies to detect Nluc and the PVM marker Exp2. DAPI was used for nuclear staining. Size bar = 5 µm.

Much of the virulence of *Plasmodium* parasites can be attributed to the export of effector proteins into the host compartment, which extensively modifies the iRBC [10], [17], [39]–[43]. Consequently, there has been much interest in understanding how parasites export proteins beyond the PVM that envelops them within the RBC cytoplasm and whether the export system can be targeted with drug inhibitors [6], [12], [39], [44]. Methods such as tagging exported proteins with fluorescent markers like green fluorescent protein or by immuno-labeling with protein-specific antibodies have been extensively applied for detection of exported proteins in *Plasmodium spp* [17], [41], [45]–[47]. Microscopy of individual parasites has then been used to follow the tagged proteins but this is time consuming and not amenable to quantification. In order to create a more quantitative approach we attempted to export Nluc into the RBC compartment so we could quantitatively follow its passage. To do this the first 113 residues of the *P. falciparum* exported protein Hyp1 (PF3D7_0113300), containing the PEXEL cleavage site RLLTE, was fused to Nluc at its N-terminus (Fig. 3A). PEXEL-Nluc parasites were analyzed by immunofluorescence (IFA) with antibodies for Nluc and Exp2, a PVM marker used to delimit the parasite boundaries. PEXEL-Nluc did not concentrate and co-localize with Exp2 rather occupying the entire RBC cytosolic region that lies beyond the PV, suggesting it is correctly exported (Fig. 3A). There was also an increased Nluc signal surrounding the parasites’ nuclei that could represent newly synthesized Nluc transiting through the endoplasmic reticulum (ER).

Once trafficked from the ER to the parasite surface probably by vesicular transport, exported proteins must transit across the PV before engaging the PVM-spanning export machine PTEX, to cross the vacuole membrane [12]. Any inhibitor of protein export is likely to trap cargo within the parasite or its vacuole and so a control parasite line was created that secreted its cargo into the PV. To do this the first 23 residues of merozoite surface protein 1 (MSP1) was fused to the N-terminus of Nluc (Fig. 3B). This region comprising the signal peptide region (SP) of MSP1 has been reported to efficiently target GFP to the secretory pathway [48]. Parasites expressing SP-Nluc were analyzed by immunofluorescence with anti-Nluc and anti-Exp2 and both proteins seem to mostly co-localize in the PV with some minor labeling in the RBC compartment (Fig. 3B).

In order to demonstrate that Nluc trafficking was dependent on the signals fused to its N-terminal region, the sub-cellular localization was also analyzed in parasites expressing the original Nluc and as expected, the reporter alone has a cytosolic localization (Fig. 3C).

### Quantification of Nluc fusions on iRBC sub-cellular compartments

Before quantifying the export of Nluc, we needed to ensure that hemoglobin a powerful quencher of luminescence detection would not mislead interpretations of the reporter activity quantified in different compartments of the infected RBCs. In order to find conditions where hemoglobin did not substantially block luminescence, PEXEL-Nluc parasites at 1% parasitemia and 10% hematocrit (ie, 10% RBCs in RPMI v/v) were sequentially diluted in RPMI. The infected RBCs were then lysed in an equal volume of Nano-Glo Luciferase Assay Reagent and the luminescence measured. After normalizing the RLU for parasite number that we have shown are directly proportional (Fig. 2B), reporter activity was similar from hematocrits of 0.04% to 0.12% (Figure S4). Hematocrits of ≥0.37% showed progressively reduced luminescence, suggesting a hemoglobin quenching effect at higher hematocrits (Figure S4). Thus for all subsequent experiments, cells extracts were always diluted so that the equivalent hematocrit was never higher than 0.1%.

**Figure 4:**
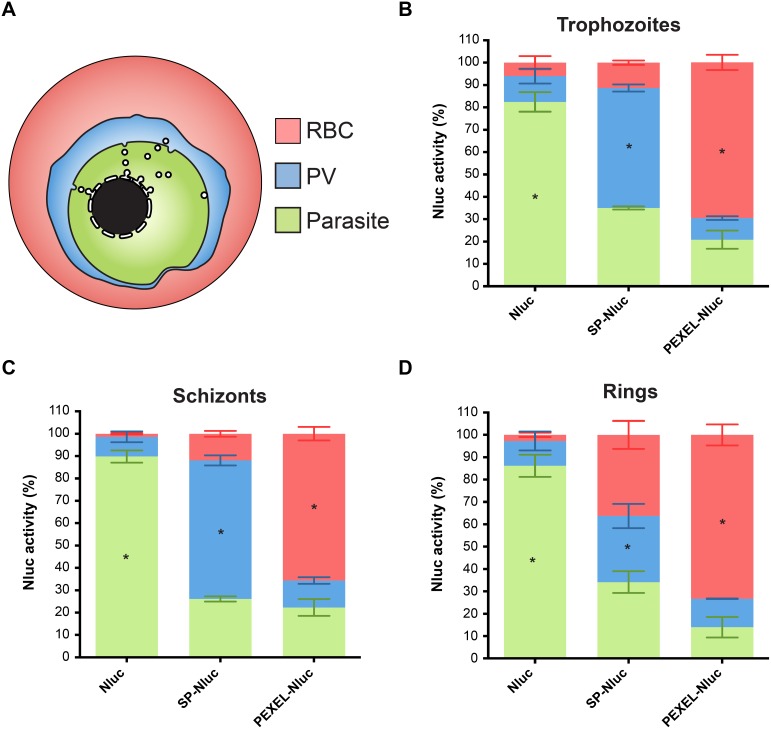
Quantification of Nluc in cellular compartments of infected RBCs. (A) Schematic of the iRBC’s compartments where RBC represents the exported fraction that is released after Equinatoxin treatment. The PV compartment was then released following treatment with 0.01% saponin and finally, the Parasite fraction was lysed by Nano-Glo Luciferase Assay Reagent. (B) Trophozoite stage parasites transfected with either the original Nluc, secreted SP-Nluc or the exported PEXEL-Nluc fusions were fractionated as shown in (A) and luciferase activity measured. Nluc activities as a percentage of the total for each parasite line are shown and represent the mean of 3 experiments +/−SEM. Similar to (B), (C) Schizonts and (D) Ring stage parasites were also fractionated. Statistical significance (* p<0.05) was determined by 2 way ANOVA test comparing the percentage of reporter activity of each sub-cellular fraction among the 3 cell lines.

Microscopy of the secreted and exported Nluc parasites indicated the fusion proteins were most strongly detected in trophozoite and schizont stage parasites (≥24 hours post invasion, [hpi]), because the EF (elongation factor 1α) promoter is weaker in younger ring stage parasites (0–24 hpi). This was confirmed by a time course luciferase assay over a single cell cycle (Figure S5) and for this reason, fractionation of the lines expressing the cytosolic Nluc, SP-Nluc and PEXEL-Nluc were first carried out on trophozoites. The subcellular compartments were first fractionated by treating the cultures with equinatoxin which forms pores in the RBC membrane, releasing the contents of the RBC cytosol where soluble exported proteins should reside [38]. The cells were then incubated with the detergent saponin, which forms pores in the PVM, releasing the PV fraction, leaving RBC and PV free parasites, which corresponds to the remaining parasite fraction (Fig. 4A). Equivalent volumes of the RBC, PV and parasite fractions from equal number of cells were then mixed with Nano-Glo Luciferase Assay Reagent and their RLU was measured.

In parasites expressing the cytosolic Nluc, more than 80% of reporter activity was detected in the parasite fraction (Fig. 4B). The remaining activity measured in the PV and RBC fractions may be caused by membrane damage during fractionation. Importantly, the percentage of activity detected in the parasite fraction of SP-Nluc and PEXEL-Nluc lines were significantly lower than in the Nluc line.

In SP-Nluc parasites just over half of the reporter activity was in the PV fraction, which was significantly higher than the PV fractions of the Nluc and PEXEL-Nluc parasites (Fig. 4B). Unexpectedly, about a third of the SP-Nluc luciferase activity was detected in the parasite fraction, which could possibly be newly synthesized enzyme en route to the parasite surface via the ER. About 10% of SP-Nluc activity was detected in the RBC fraction, which may be due to membrane damage or leakage out of the vacuole prior to fractionation. Alternatively, the small amount of NLuc activity detected in the RBC fraction may reflect the microscopy results from earlier indicating that in some SP-Nluc expressing parasites, the RBC cytosol was also labeled with anti-NLuc in addition to the PV (Fig. 3B).

About 70% of luciferase activity was detected in the RBC fraction of PEXEL-Nluc parasites (Fig. 4B), which is substantially higher than in Nluc and SP-Nluc lines. The higher reporter activity in the Parasite fraction in the PEXEL-Nluc parasites compared to the PV fraction could indicate Nluc takes longer to be secreted from the parasite than it does to transit the PV. Taken together these results indicate that Nluc fusion proteins are targeted correctly to different cell compartments and can be used to investigate and to quantify cellular trafficking.

While the secretory pathway is clearly functioning during schizogony, little is know about the ability of parasites to export proteins during this stage, bringing into question whether PEXEL-Nluc could be exported or would remain trapped in the PV. Cellular fractionation was therefore carried out with segmented schizonts and the pattern was very similar to trophozoites (Fig. 4C). The fact that total luciferase activity in these lines increases until at least 32 hpi (Figure S5) and that a substantial part of the RBC is engulfed by the parasites strongly suggest PEXEL-Nluc is exported in schizonts.

Despite the weakness of the EF-1α promoter in rings (Figure S5), we attempted RBC fractionation and found the cytosolic and exported lines had a pattern very similar to trophozoites and schizonts (Fig. 4D). In rings however, the secreted line had higher proportion of reporter activity in the RBC fraction than in older trophozoites and schizonts (Fig. 4B–D). The proportion of signal in the SP-Nluc Parasite fraction was not altered, but in the PV fraction it had been reduced. It is possible that the ring-stage PVM is more susceptible to some degree of equinatoxin permeabilisation than in mature stages, but given the very low expression of SP-Nluc in rings, there could be an experimental artifact such as some leakage of SP-Nluc into the RBC during invasion when the PVM forms. Despite a higher proportion of reporter activity in the RBC fraction in SP-Nluc ring stages, this line still has the signature of a secreted reporter. The luciferase activity in the PV fraction is still substantially higher than in the equivalent fraction in the cytosolic and exported lines (Fig. 4D). In addition, reporter activity in the RBC fraction of SP-Nluc rings is considerably lower than in PEXEL-Nluc rings (Fig. 4D).

### Inhibition by Brefeldin A

**Figure 5:**
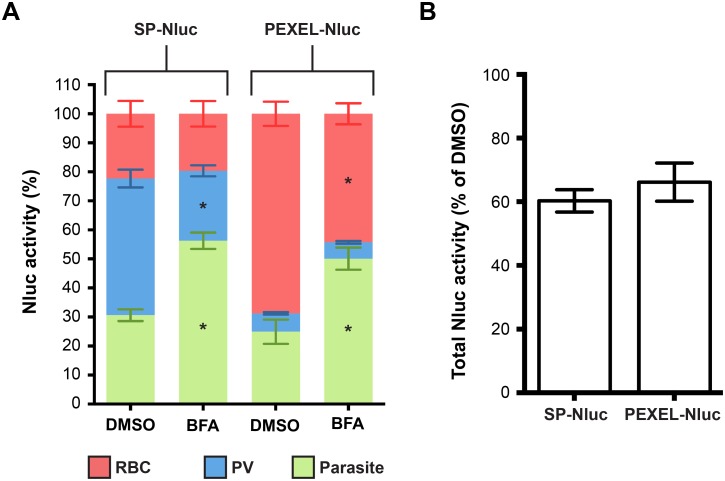
Detection of secretion and export inhibition. (A) Parasites expressing the secreted (SP) or the exported (PEXEL) Nluc were treated with Brefeldin A (BFA) for 6 hours and luciferase activity determined in each sub-cellular fraction. Results are the mean of 4 experiments and the error bars represent standard deviations. Statistical significance (* p<0.05) was determined by 2 way ANOVA test comparing the activity detected in the parasite fraction of treated and DMSO control parasites of each line and of PV and RBC fractions of DMSO against treated SP-Nluc and PEXEL-Nluc lines respectively. (B) The toxicity of each treatment was determined by measuring total luciferase activity as relative to the DMSO control.

With the Nluc, SP-Nluc and PEXEL-Nluc parasites having such distinct and characteristic reporter fraction patterns, we decided to test known trafficking inhibitors on the lines to assess their potential usefulness for screening for novel trafficking inhibitor compounds. To our knowledge, however there are no known *Plasmodium*-specific protein export inhibitors, so we instead used the antibiotic brefeldin A (BFA), which blocks the translocation of proteins from the ER to the Golgi. Since this pathway is common for both protein secretion and export, SP-Nluc and PEXEL-Nluc lines were treated with BFA followed by RBC fractionation (Fig. 5A). Treatment of trophozoites with 5 mg/mL BFA for 6 hrs in both lines caused an increase in reporter activity in the parasite suggesting luciferase is accumulating in the parasite as expected. In the SP-Nluc line, activity decreased in the PV fraction but not in the RBC, since transit from the PV into the RBC was not expected to be affected by BFA. In the PEXEL-Nluc line, activity was reduced in the RBC, but no change was seen in the PV fraction suggesting that BFA does not block the PTEX protein transporter. The general BFA toxicity to the cells was evaluated by measuring total reporter activity and it dropped by 40 and 35% for the SP-Nluc and PEXEL-Nluc lines respectively, during the assay period (Fig. 5B).

### Inhibition of New Permeation Pathways

**Figure 6:**
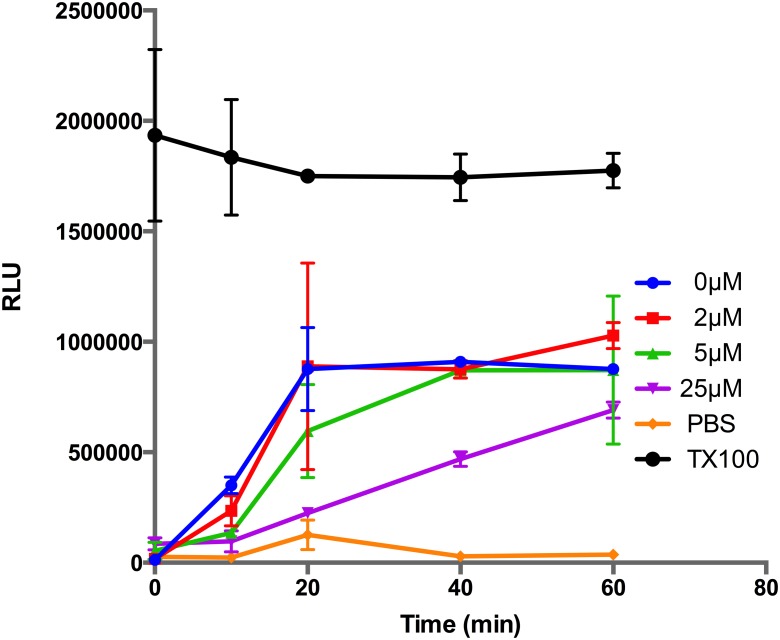
Quantification of NPP activity and inhibition in PEXEL-NLuc parasites. Trophozoite stage parasites (3% parasitemia) expressing exported PEXEL-NLuc were incubated with different concentrations of the new permeability pathway (NPP) inhibitor furosemide (µM), and then treated with sorbitol lysis buffer at time zero to induce the release of PEXEL-Nluc which was measured over time. A 1% Triton-X100 in PBS (TX100) treatment was included to simulate rapid lysis and a PBS control for background lysis. Data points represent the mean +/− SD of three replicates.

As the parasite matures from a ring into a trophozoite it progressively increases the permeability of the iRBC to acquire plasma nutrients and remove waste products that are vital for rapid growth. The increased permeability appears to be due to the formation of anion selective channels in the iRBC plasma membrane. While its clear that parasites play a role in generating the NPPs, it is not known if they directly form the membrane channels *de novo* or if they increase the conductance of pre-existing human channels [11], [49]. It has long been recognised that if NPPs could be blocked with drug inhibitors then this may starve the parasites severely curtailing their growth. Screens for such inhibitors have been based on the susceptibility of iRBCs to an iso-osmotic sorbitol solution that lyses mature parasites with functional NPPs [50], [51]. Compounds that block NPPs render the parasites resistant to the sorbitol and the resultant reduction in haemoglobin from lysed iRBCs is measured. To obtain sufficient sensitivity, large numbers of purified iRBC have to be assayed and we therefore wished to determine whether exported PEXEL-Nluc from lysed iRBCs might be more sensitively detected, and could be quantified without prior isolation of the iRBCs from uninfected RBCs. To test this, PEXEL-Nluc trophozoite-stage parasites at 3–5% parasitemia were incubated with the benchmark NPP inhibitor furosemide [50] prior to addition of the sorbitol solution containing Nano-Glo. Bioluminescence produced by the released PEXEL-Nluc was measured as a function of time and it was observed that without furosemide pre-treatment (0 µM), sorbitol triggered high Nluc activity that peaked 20 minutes after the addition of sorbitol (Fig. 6). Pre-treatment with furosemide greatly reduced the release of PEXEL-Nluc in a concentration dependent manner (Fig. 6). In order to determine the sorbitol incubation period that would allow optimal detection of NPP inhibition, robustness parameters were calculated for 10–60 min times (Table 2). While the shortest time (10 min) produces an acceptable Z’ value, ≥0.5, 20–40 min appeared to be the most suitable incubation period, where the Z’ value was the highest. At 60 min, iRBC tended to burst even in the presence of the highest concentration of furosemide, suggesting the NPP inhibition is partial, resulting in lower S/B and S/N ratios and a highly variable Z’ value. The benefit of PEXEL-Nluc parasites is the sensitivity of the assay which requires no parasite enrichment and hence fewer parasites and manipulations compared to previous methods rendering the assay more suitable for high throughput screening.

**Table 2:** Summary of the assay parameters from the NPP assay.

	Incubation time			
	10 min	20 min	40 min	60 min
**Z’-value**	0.65±0.19	0.80±0.12	0.81±0.02	−0.20±1.24
**S/B ratio**	2.7±0.19	4.83±1.96	2.72±0.49	1.47±0.11
**S/N ratio**	50.6±13.3	54.84±27.54	37.28±10.91	10.49±11.09
**%CV_max_**	5.94±3.33	3.76±2.79	2.33±1.22	5.33±4.92
**%CV_min_**	3.43±0.53	9.01±8.09	4.61±0.04	8.87±8.28

## Discussion

*P. falciparum* bioluminescent lines have been widely used both in studies of parasite biology as well as in assays where detection and quantification of parasite development or maturation is required [20]–[22], [25], [27]. However, this technology has some caveats such as the low transfection efficiency and consequently the long periods required to generate stable transfectants. Here we have shown that the significantly brighter Nluc reporter can overcome some of these difficulties. To begin with we have shown that transiently transfected parasites can be used for drug assays, opening the possibility of assaying drug sensitivity/resistance in many parasite strains and field isolates without having to generate stable lines. The absence of species barrier for promoter recognition in *Plasmodium* [52], [53] suggests this assay could be applied for parasites of other species, including *P. vivax*, a parasite for which *in vitro* culturing is more complex and less affordable. Our experimental design involved two cycles of RBC invasion; the first when RBCs electroporated with plasmid were added to mature stage parasites; and the second when parasites have grown in the presence of chloroquine for 3 days. To overcome poor invasion efficiency in newly cultured *P. falciparum* clinical isolates and of *P. vivax*, transient transfection could be performed by electroporating ring stage parasites and measuring reporter activity as parasites mature. Previous results have shown that luciferase based assays are suitable for monitoring parasite maturation and the effect of fast acting drugs [26], [27].

Although funds for malaria control and eradication have increased in the last decade, they are still less than half of the U$5 billion that is estimated to be required [1]. Since drug discovery is an essential part of control/eradication strategies, reducing the cost of screening cultured parasites to discover new inhibitors is highly desirable. When used at its recommended concentration the cost of the Nano-Glo reagent for screening a 96 well plate of Nluc expressing *P. falciparum* parasites is several dollars. However, we have shown that the Nano-Glo reagent can be diluted a 1,000-fold while still maintaining sufficient sensitivity. This could be suitable for high throughput screening and represent an attractive option to researchers with limited resources. In addition, the stability of Nluc in frozen samples makes it practical for storage and transport to where the Nluc can be measured. In our current drug assays, parasite cultures were kept frozen at −80°C for several weeks or at −20°C for several days without any loss of reporter activity that would affect the results (data not shown).

Protein export plays an essential role for *P. falciparum* infection where it is strongly associated with virulence and severity of disease. Investigation of exported protein trafficking has relied on examination by fluorescence microscopy of protein fusions with fluorescent markers or labelling with fluorescent antibodies. Such techniques require highly trained personnel as well as expensive and sophisticated microscopes. Microscopy is also poorly quantitative since only a relatively small number of cells can be practically examined. Bioluminescence offers a simpler and more quantifiable method to measure protein export, requiring less training and significantly more affordable equipment. We have established a protocol for the small-scale quantification of protein secretion and export in stable Nluc transfected parasites that can be used in tandem with general drug screening protocols to evaluate the effect on export of compounds previously shown to inhibit parasite growth. Since there are no known specific protein export inhibitors, we could not test whether PEXEL-Nluc expressing parasites could be used to screen for export inhibition. Instead, BFA treatment that blocks transit from ER to Golgi was used to confirm that inhibition of protein export and secretion can be quantified. The fact that export is similarly BFA-inhibited as secretion confirms both trafficking systems share common steps in the secretory pathway [46], [47], [54], [55].

The Nluc fusion proteins generally appear to localize where they were expected with data from both microscopy and reporter activity of sub-cellular fractionation suggesting a small leakage of SP-Nluc from the PV to RBC. It is known that the PVM looses integrity and becomes leaky in late schizonts [56], which could have allowed for the small soluble SP-Nluc (19 kDa) to be detected in the RBC fraction. Strong leakage in rings indicates that the PVM could be more permeable than previously thought or just more susceptible to equinatoxin damage. Despite this, the cytoplasmic, vacuolar and exported parasite Nluc lines once fractionated, have unique signatures that would enable screening for inhibitors that prevent growth, secretion and export. Our microtube cell fractionation protocol is currently not suitable for the high throughput screening of export inhibitors in microplate format due to the large number of steps needed to fractionate the cell compartments. We are however continuing to improve the protocol including its adaption for quantifying export in transiently transfected parasites.

In contrast to our assay for protein export inhibition, the NPP inhibition assay seems considerably more robust and ready to be adapted for high throughput screening. The PEXEL-Nluc NPP assay has the advantage over the existing protocol, which measures haemoglobin release, in that the iRBCs do not need to be purified. Moreover, due it its higher sensitivity, at least 50 times fewer parasites are needed which is better suited for large scale screening. In conclusion, we have shown that the use of the new Nluc reporter in *P. falciparum* has significant advantages when compared to the most commonly used firefly luciferase, offering simple and affordable assays to quantify growth and cellular trafficking to iRBC compartments.

## Supporting Information

Figure S1 **Graph of raw RLU vs parasitemia of same data presented in** **Fig. 2C** **.**
(TIF)

Figure S2 **Graph of raw RLU vs iRBC number.** (A) Samples and conditions are the same as in Fig. 2C, except that gain was adjusted to maximum. (B) Samples were measured for 10 sec, gain was set to maximum and Nano-Glo was used in its standard dilution.
(TIF)

Figure S3 **Graph of raw RLU vs iRBC number. PEXEL-Nluc parasites were serially diluted and samples measured as in** **Fig. 2C** **.**
(TIF)

Figure S4 **Hemoglobin quenches bioluminescence detection.** Nluc expressing parasites were diluted in RPMI in 3-fold increments and luciferase activity determined. Values were normalized by parasite number and represented relative to the lowest hematocrit (0.04%). The bars represent the mean ± standard deviation of 4 experiments.
(TIF)

Figure S5 **Nluc stably expressed from the pEF vector shows maximal expression during the last half of the cell cycle.** To tightly synchronise the PEXEL-Nluc parasites so that the bioluminescence could be accurately measured during the cell cycle, heparin was added to schizonts prior to them rupturing. The heparin inhibits invasion and was removed once the schizonts began rupturing and then added back 2 hours later to produce an invasion window of 2 hours. The remaining unruptured schizonts were lysed in 5% sorbitol. The synchronized parasites were harvested every 4 hours until the 32–34 hour time point. Cultures were then diluted to 0.1% hematocrit and added to 1 volume of Nano-Glo Luciferase Assay Reagent and Nluc activity measured. Nluc values are represented as % of the maximum activity. Hours post invasion values are the mid-point of the 2 hour window.
(TIF)

## References

[1] WHO (2013) World malaria report 2013.

[2] Gamo FJ, Sanz LM, Vidal J, de Cozar C, Alvarez E, et al (2010) Thousands of chemical starting points for antimalarial lead identification. Nature 465: 305–310. 20485427 10.1038/nature09107

[3] Guiguemde WA, Shelat AA, Bouck D, Duffy S, Crowther GJ, et al (2010) Chemical genetics of Plasmodium falciparum. Nature 465: 311–315. 20485428 10.1038/nature09099 PMC2874979

[4] Lucumi E, Darling C, Jo H, Napper AD, Chandramohanadas R, et al (2010) Discovery of potent small-molecule inhibitors of multidrug-resistant Plasmodium falciparum using a novel miniaturized high-throughput luciferase-based assay. Antimicrob Agents Chemother 54: 3597–3604. 20547797 10.1128/AAC.00431-10 PMC2934977

[5] Spangenberg T, Burrows JN, Kowalczyk P, McDonald S, Wells TN, et al (2013) The open access malaria box: a drug discovery catalyst for neglected diseases. PLoS One 8: e62906. 23798988 10.1371/journal.pone.0062906 PMC3684613

[6] Boddey JA, Hodder AN, Gunther S, Gilson PR, Patsiouras H, et al (2010) An aspartyl protease directs malaria effector proteins to the host cell. Nature 463: 627–631. 20130643 10.1038/nature08728 PMC2818761

[7] Marti M, Spielmann T (2013) Protein export in malaria parasites: many membranes to cross. Curr Opin Microbiol 16: 445–451. 23725671 10.1016/j.mib.2013.04.010 PMC3755040

[8] Pasini EM, Braks JA, Fonager J, Klop O, Aime E, et al (2013) Proteomic and genetic analyses demonstrate that Plasmodium berghei blood stages export a large and diverse repertoire of proteins. Mol Cell Proteomics 12: 426–448. 23197789 10.1074/mcp.M112.021238 PMC3567864

[9] Maier AG, Cooke BM, Cowman AF, Tilley L (2009) Malaria parasite proteins that remodel the host erythrocyte. Nat Rev Microbiol 7: 341–354. 19369950 10.1038/nrmicro2110

[10] Maier AG, Rug M, O'Neill MT, Brown M, Chakravorty S, et al (2008) Exported proteins required for virulence and rigidity of Plasmodium falciparum-infected human erythrocytes. Cell 134: 48–61. 18614010 10.1016/j.cell.2008.04.051 PMC2568870

[11] Nguitragool W, Bokhari AA, Pillai AD, Rayavara K, Sharma P, et al (2011) Malaria parasite clag3 genes determine channel-mediated nutrient uptake by infected red blood cells. Cell 145: 665–677. 21620134 10.1016/j.cell.2011.05.002 PMC3105333

[12] de Koning-Ward TF, Gilson PR, Boddey JA, Rug M, Smith BJ, et al (2009) A newly discovered protein export machine in malaria parasites. Nature 459: 945–949. 19536257 10.1038/nature08104 PMC2725363

[13] Beck JR, Muralidharan V, Oksman A, Goldberg DE (2014) PTEX component HSP101 mediates export of diverse malaria effectors into host erythrocytes. Nature 511: 592–595. 25043010 10.1038/nature13574 PMC4130291

[14] Elsworth B, Matthews K, Nie CQ, Kalanon M, Charnaud SC, et al (2014) PTEX is an essential nexus for protein export in malaria parasites. Nature 511: 587–591. 25043043 10.1038/nature13555

[15] Adjalley SH, Johnston GL, Li T, Eastman RT, Ekland EH, et al (2011) Quantitative assessment of Plasmodium falciparum sexual development reveals potent transmission-blocking activity by methylene blue. Proc Natl Acad Sci U S A 108: E1214–1223. 22042867 10.1073/pnas.1112037108 PMC3223476

[16] Lucantoni L, Duffy S, Adjalley SH, Fidock DA, Avery VM (2013) Identification of MMV malaria box inhibitors of plasmodium falciparum early-stage gametocytes using a luciferase-based high-throughput assay. Antimicrob Agents Chemother 57: 6050–6062. 24060871 10.1128/AAC.00870-13 PMC3837862

[17] Marti M, Good RT, Rug M, Knuepfer E, Cowman AF (2004) Targeting malaria virulence and remodeling proteins to the host erythrocyte. Science 306: 1930–1933. 15591202 10.1126/science.1102452

[18] Goonewardene R, Daily J, Kaslow D, Sullivan TJ, Duffy P, et al (1993) Transfection of the malaria parasite and expression of firefly luciferase. Proc Natl Acad Sci U S A 90: 5234–5236. 8506371 10.1073/pnas.90.11.5234 PMC46690

[19] Militello KT, Wirth DF (2003) A new reporter gene for transient transfection of Plasmodium falciparum. Parasitol Res 89: 154–157. 12489017 10.1007/s00436-002-0721-5

[20] Azevedo MF, del Portillo HA (2007) Promoter regions of Plasmodium vivax are poorly or not recognized by Plasmodium falciparum. Malar J 6: 20. 17313673 10.1186/1475-2875-6-20 PMC1805447

[21] Frank M, Dzikowski R, Costantini D, Amulic B, Berdougo E, et al (2006) Strict pairing of var promoters and introns is required for var gene silencing in the malaria parasite Plasmodium falciparum. J Biol Chem 281: 9942–9952. 16455655 10.1074/jbc.M513067200 PMC3941977

[22] Horrocks P, Lanzer M (1999) Mutational analysis identifies a five base pair cis-acting sequence essential for GBP130 promoter activity in Plasmodium falciparum. Mol Biochem Parasitol 99: 77–87. 10215026 10.1016/s0166-6851(98)00182-0

[23] Che P, Cui L, Kutsch O, Cui L, Li Q (2012) Validating a firefly luciferase-based high-throughput screening assay for antimalarial drug discovery. Assay Drug Dev Technol 10: 61–68. 22050430 10.1089/adt.2011.0378 PMC3277734

[24] Cui L, Miao J, Wang J, Li Q, Cui L (2008) Plasmodium falciparum: development of a transgenic line for screening antimalarials using firefly luciferase as the reporter. Exp Parasitol 120: 80–87. 18579134 10.1016/j.exppara.2008.05.003 PMC2559859

[25] Ekland EH, Schneider J, Fidock DA (2011) Identifying apicoplast-targeting antimalarials using high-throughput compatible approaches. FASEB J 25: 3583–3593. 21746861 10.1096/fj.11-187401 PMC3177575

[26] Hasenkamp S, Sidaway A, Devine O, Roye R, Horrocks P (2013) Evaluation of bioluminescence-based assays of anti-malarial drug activity. Malar J 12: 58. 23394077 10.1186/1475-2875-12-58 PMC3571881

[27] Khan T, van Brummelen AC, Parkinson CJ, Hoppe HC (2012) ATP and luciferase assays to determine the rate of drug action in in vitro cultures of Plasmodium falciparum. Malar J 11: 369. 23134617 10.1186/1475-2875-11-369 PMC3505462

[28] Burghaus PA, Lingelbach K (2001) Luciferase, when fused to an N-terminal signal peptide, is secreted from transfected Plasmodium falciparum and transported to the cytosol of infected erythrocytes. J Biol Chem 276: 26838–26845. 11375978 10.1074/jbc.M100111200

[29] Hall MP, Unch J, Binkowski BF, Valley MP, Butler BL, et al (2012) Engineered luciferase reporter from a deep sea shrimp utilizing a novel imidazopyrazinone substrate. ACS Chem Biol 7: 1848–1857. 22894855 10.1021/cb3002478 PMC3501149

[30] de Azevedo MF, Gilson PR, Gabriel HB, Simoes RF, Angrisano F, et al (2012) Systematic analysis of FKBP inducible degradation domain tagging strategies for the human malaria parasite Plasmodium falciparum. PLoS One 7: e40981. 22815885 10.1371/journal.pone.0040981 PMC3397994

[31] Trager W, Jensen JB (1976) Human malaria parasites in continuous culture. Science 193: 673–675. 781840 10.1126/science.781840

[32] Deitsch K, Driskill C, Wellems T (2001) Transformation of malaria parasites by the spontaneous uptake and expression of DNA from human erythrocytes. Nucleic Acids Res 29: 850–853. 11160909 10.1093/nar/29.3.850 PMC30384

[33] Hasenkamp S, Russell KT, Horrocks P (2012) Comparison of the absolute and relative efficiencies of electroporation-based transfection protocols for Plasmodium falciparum. Malar J 11: 210. 22720754 10.1186/1475-2875-11-210 PMC3407700

[34] Makler MT, Hinrichs DJ (1993) Measurement of the lactate dehydrogenase activity of Plasmodium falciparum as an assessment of parasitemia. Am J Trop Med Hyg 48: 205–210. 8447524 10.4269/ajtmh.1993.48.205

[35] Zhang JH, Chung TD, Oldenburg KR (1999) A Simple Statistical Parameter for Use in Evaluation and Validation of High Throughput Screening Assays. J Biomol Screen 4: 67–73. 10838414 10.1177/108705719900400206

[36] Tonkin CJ, van Dooren GG, Spurck TP, Struck NS, Good RT, et al (2004) Localization of organellar proteins in Plasmodium falciparum using a novel set of transfection vectors and a new immunofluorescence fixation method. Mol Biochem Parasitol 137: 13–21. 15279947 10.1016/j.molbiopara.2004.05.009

[37] Bullen HE, Charnaud SC, Kalanon M, Riglar DT, Dekiwadia C, et al (2012) Biosynthesis, localization, and macromolecular arrangement of the Plasmodium falciparum translocon of exported proteins (PTEX). J Biol Chem 287: 7871–7884. 22253438 10.1074/jbc.M111.328591 PMC3318755

[38] Jackson KE, Spielmann T, Hanssen E, Adisa A, Separovic F, et al (2007) Selective permeabilization of the host cell membrane of Plasmodium falciparum-infected red blood cells with streptolysin O and equinatoxin II. Biochem J 403: 167–175. 17155936 10.1042/BJ20061725 PMC1828889

[39] Boddey JA, Carvalho TG, Hodder AN, Sargeant TJ, Sleebs BE, et al (2013) Role of plasmepsin V in export of diverse protein families from the Plasmodium falciparum exportome. Traffic 14: 532–550. 23387285 10.1111/tra.12053

[40] Heiber A, Kruse F, Pick C, Gruring C, Flemming S, et al (2013) Identification of new PNEPs indicates a substantial non-PEXEL exportome and underpins common features in Plasmodium falciparum protein export. PLoS Pathog 9: e1003546. 23950716 10.1371/journal.ppat.1003546 PMC3738491

[41] Hiller NL, Bhattacharjee S, van Ooij C, Liolios K, Harrison T, et al (2004) A host-targeting signal in virulence proteins reveals a secretome in malarial infection. Science 306: 1934–1937. 15591203 10.1126/science.1102737

[42] Sargeant TJ, Marti M, Caler E, Carlton JM, Simpson K, et al (2006) Lineage-specific expansion of proteins exported to erythrocytes in malaria parasites. Genome Biol 7: R12. 16507167 10.1186/gb-2006-7-2-r12 PMC1431722

[43] van Ooij C, Tamez P, Bhattacharjee S, Hiller NL, Harrison T, et al (2008) The malaria secretome: from algorithms to essential function in blood stage infection. PLoS Pathog 4: e1000084. 18551176 10.1371/journal.ppat.1000084 PMC2408878

[44] Elsworth B, Crabb BS, Gilson PR (2014) Protein export in malaria parasites: an update. Cell Microbiol 16: 355–363. 24418476 10.1111/cmi.12261

[45] Boddey JA, Moritz RL, Simpson RJ, Cowman AF (2009) Role of the Plasmodium export element in trafficking parasite proteins to the infected erythrocyte. Traffic 10: 285–299. 19055692 10.1111/j.1600-0854.2008.00864.x PMC2682620

[46] Knuepfer E, Rug M, Klonis N, Tilley L, Cowman AF (2005) Trafficking of the major virulence factor to the surface of transfected P. falciparum-infected erythrocytes. Blood 105: 4078–4087. 15692070 10.1182/blood-2004-12-4666 PMC1895071

[47] Wickham ME, Rug M, Ralph SA, Klonis N, McFadden GI, et al (2001) Trafficking and assembly of the cytoadherence complex in Plasmodium falciparum-infected human erythrocytes. EMBO J 20: 5636–5649. 11598007 10.1093/emboj/20.20.5636 PMC125667

[48] Gilson PR, O'Donnell RA, Nebl T, Sanders PR, Wickham ME, et al (2008) MSP1(19) miniproteins can serve as targets for invasion inhibitory antibodies in Plasmodium falciparum provided they contain the correct domains for cell surface trafficking. Mol Microbiol 68: 124–138. 18333885 10.1111/j.1365-2958.2008.06140.x

[49] Bouyer G, Cueff A, Egee S, Kmiecik J, Maksimova Y, et al (2011) Erythrocyte peripheral type benzodiazepine receptor/voltage-dependent anion channels are upregulated by Plasmodium falciparum. Blood 118: 2305–2312. 21795748 10.1182/blood-2011-01-329300

[50] Duranton C, Huber SM, Tanneur V, Brand VB, Akkaya C, et al (2004) Organic osmolyte permeabilities of the malaria-induced anion conductances in human erythrocytes. J Gen Physiol 123: 417–426. 15051807 10.1085/jgp.200308919 PMC2217455

[51] Huber SM, Uhlemann AC, Gamper NL, Duranton C, Kremsner PG, et al (2002) Plasmodium falciparum activates endogenous Cl(−) channels of human erythrocytes by membrane oxidation. EMBO J 21: 22–30. 11782422 10.1093/emboj/21.1.22 PMC125814

[52] Crabb BS, Cowman AF (1996) Characterization of promoters and stable transfection by homologous and nonhomologous recombination in Plasmodium falciparum. Proc Natl Acad Sci U S A 93: 7289–7294. 8692985 10.1073/pnas.93.14.7289 PMC38976

[53] Fernandez-Becerra C, de Azevedo MF, Yamamoto MM, del Portillo HA (2003) Plasmodium falciparum: new vector with bi-directional promoter activity to stably express transgenes. Exp Parasitol 103: 88–91. 12810052 10.1016/s0014-4894(03)00065-1

[54] Ansorge I, Benting J, Bhakdi S, Lingelbach K (1996) Protein sorting in Plasmodium falciparum-infected red blood cells permeabilized with the pore-forming protein streptolysin O. Biochem J 315 (Pt. 1): 307–314. 10.1042/bj3150307 PMC1217187 8670123

[55] Chang HH, Falick AM, Carlton PM, Sedat JW, DeRisi JL, et al (2008) N-terminal processing of proteins exported by malaria parasites. Mol Biochem Parasitol 160: 107–115. 18534695 10.1016/j.molbiopara.2008.04.011 PMC2922945

[56] Collins CR, Hackett F, Strath M, Penzo M, Withers-Martinez C, et al (2013) Malaria parasite cGMP-dependent protein kinase regulates blood stage merozoite secretory organelle discharge and egress. PLoS Pathog 9: e1003344. 23675297 10.1371/journal.ppat.1003344 PMC3649973

