# Supplementary figures and images for: Plasmodium falciparum Transfected with Ultra Bright NanoLuc Luciferase Offers High Sensitivity Detection for the Screening of Growth and Cellular Trafficking Inhibitors
Source: PLoS One. 2014 Nov 13;9(11):e112571. doi: 10.1371/journal.pone.0112571 (PMC4231029; doi:10.1371/journal.pone.0112571)

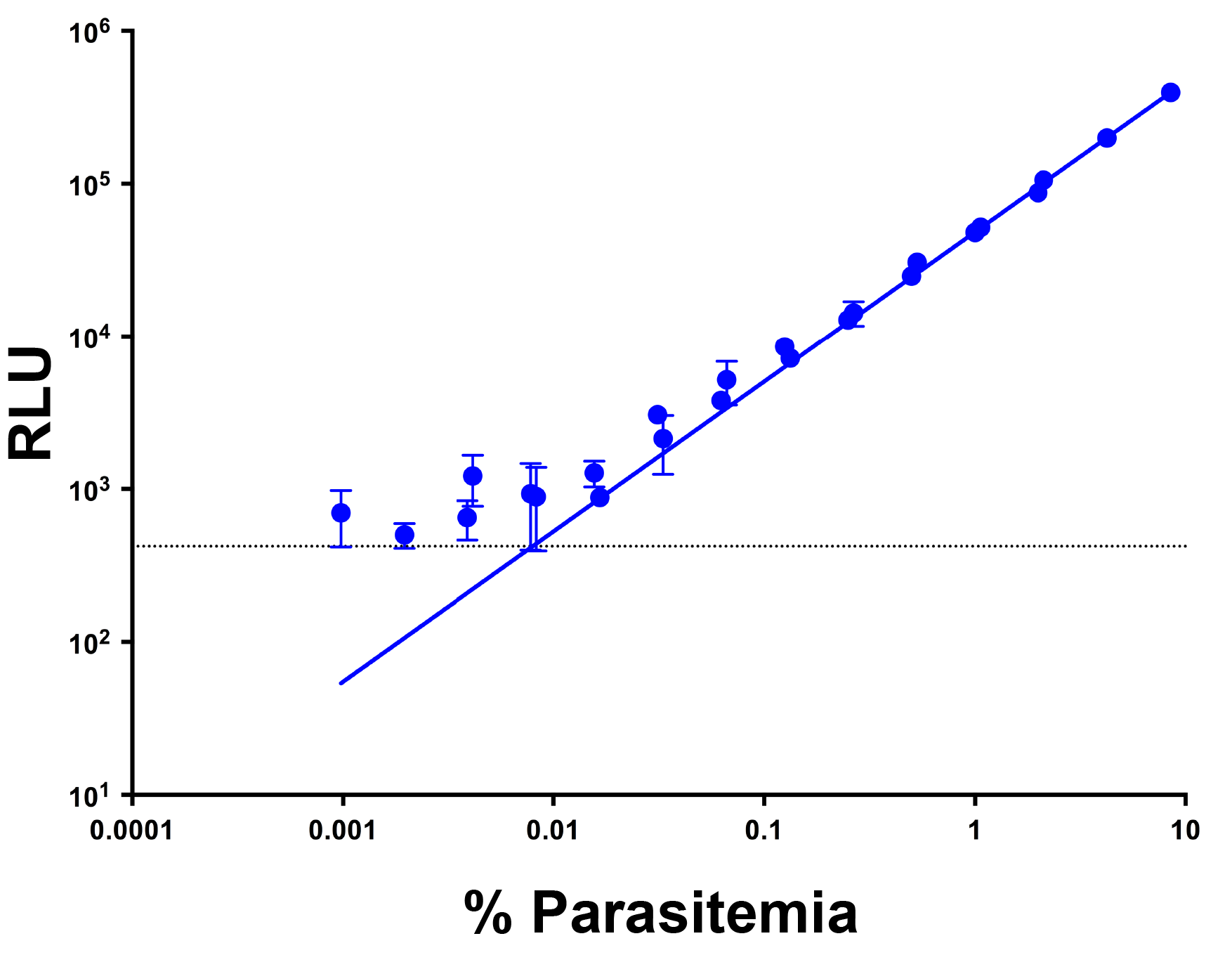

Supplement: Figure S1 — Graph of raw RLU vs parasitemia of same data presented in Fig. 2C . (TIF) [file pone.0112571.s001.tif]

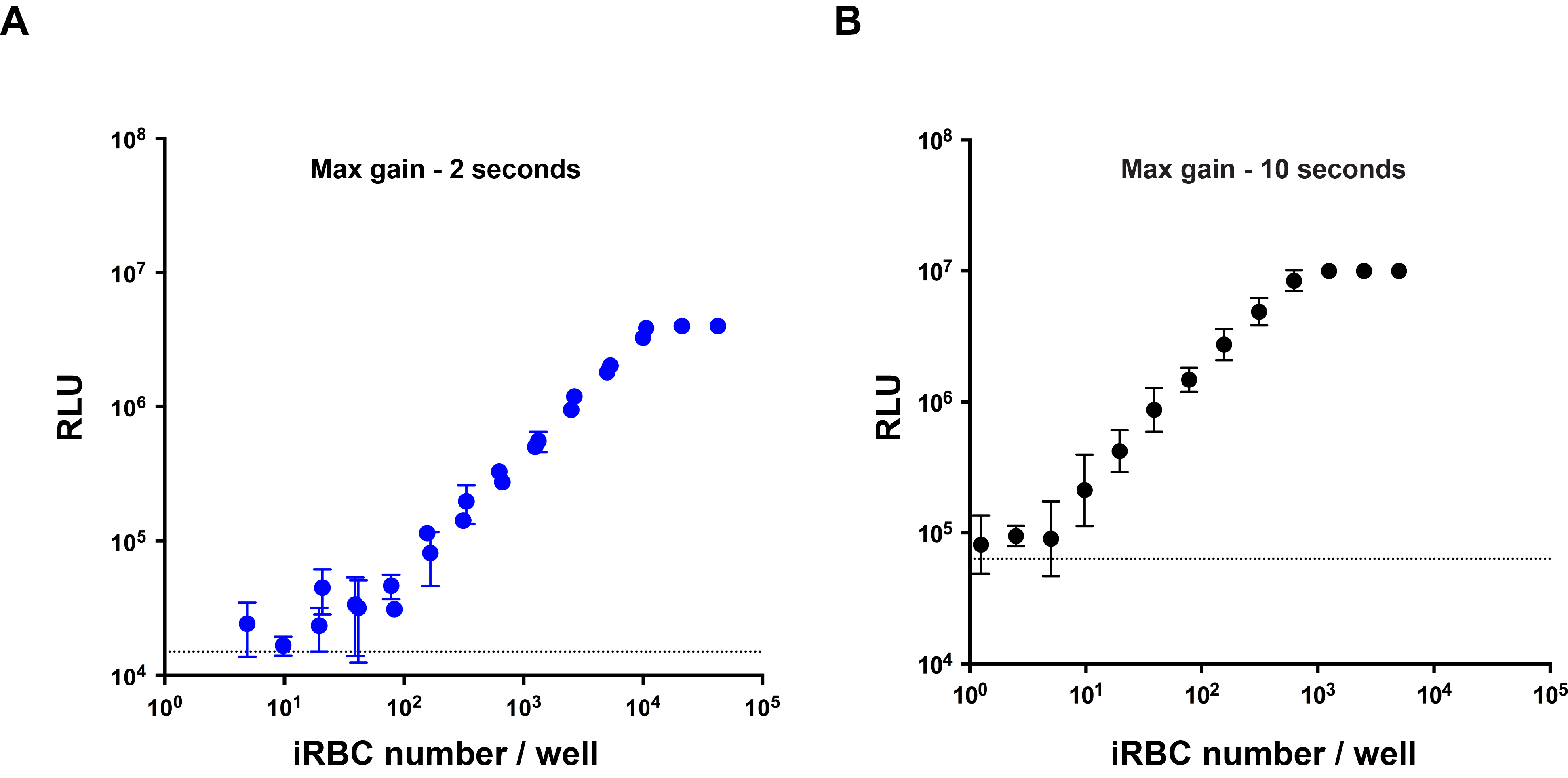

Supplement: Figure S2 — Graph of raw RLU vs iRBC number. (A) Samples and conditions are the same as in Fig. 2C, except that gain was adjusted to maximum. (B) Samples were measured for 10 sec, gain was set to maximum and Nano-Glo was used in its standard dilution. (TIF) [file pone.0112571.s002.tif]

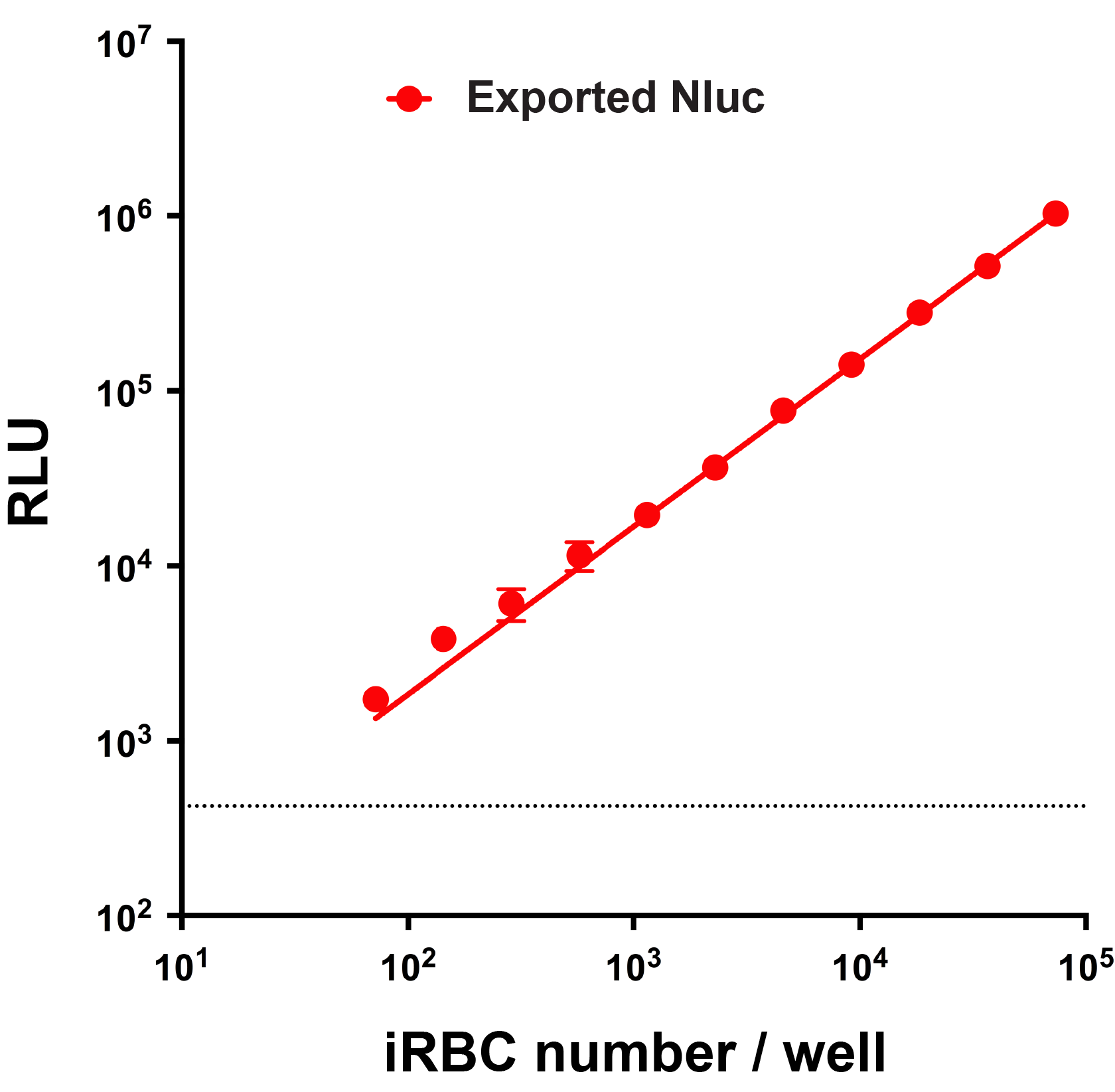

Supplement: Figure S3 — Graph of raw RLU vs iRBC number. PEXEL-Nluc parasites were serially diluted and samples measured as in Fig. 2C . (TIF) [file pone.0112571.s003.tif]

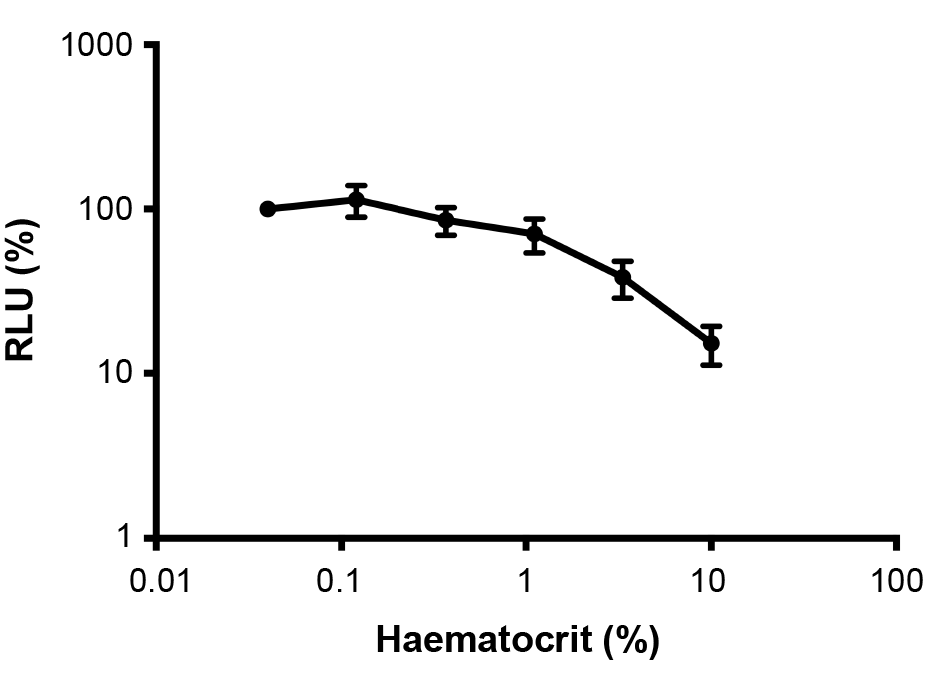

Supplement: Figure S4 — Hemoglobin quenches bioluminescence detection. Nluc expressing parasites were diluted in RPMI in 3-fold increments and luciferase activity determined. Values were normalized by parasite number and represented relative to the lowest hematocrit (0.04%). The bars represent the mean ± standard deviation of 4 experiments. (TIF) [file pone.0112571.s004.tif]

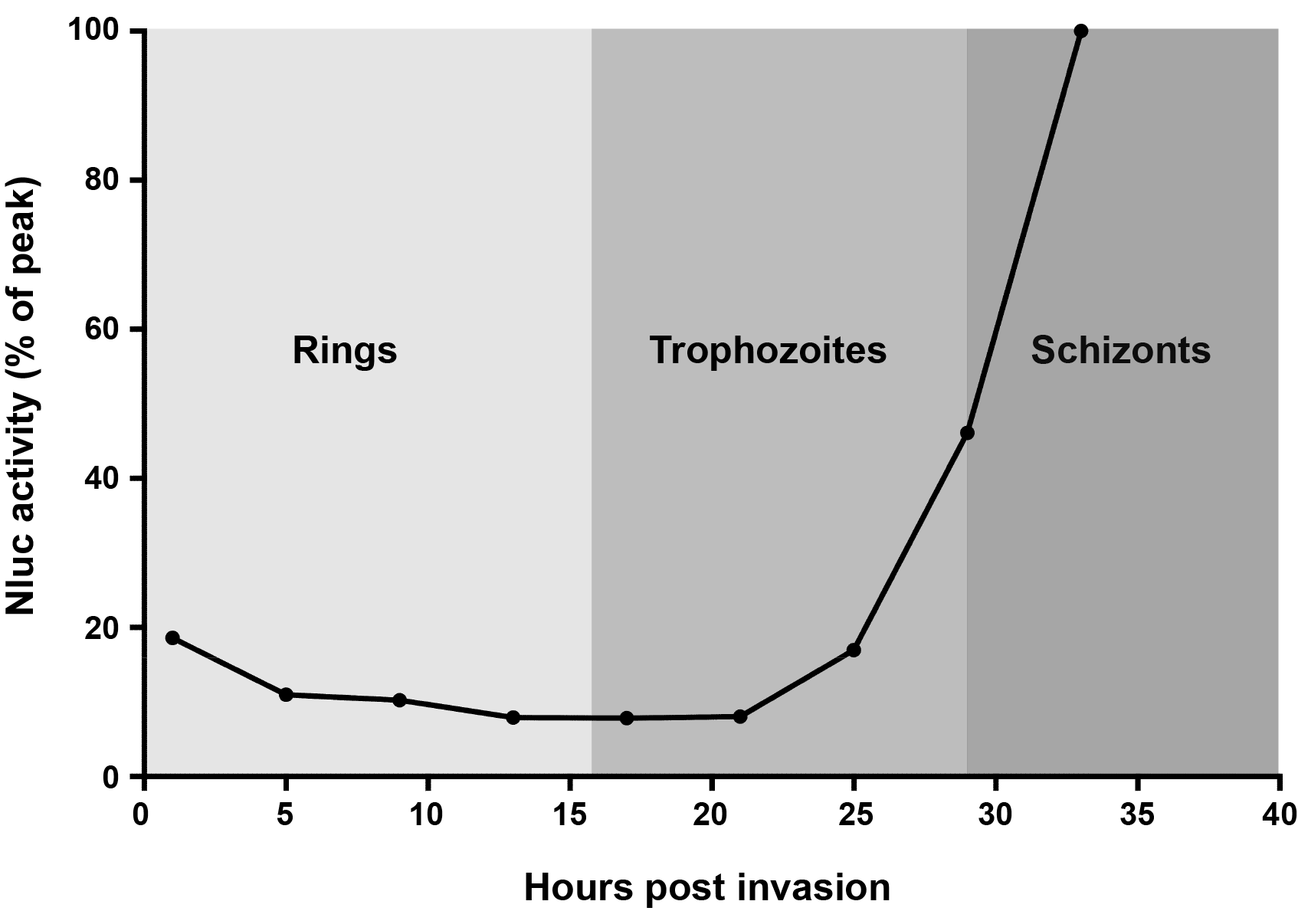

Supplement: Figure S5 — Nluc stably expressed from the pEF vector shows maximal expression during the last half of the cell cycle. To tightly synchronise the PEXEL-Nluc parasites so that the bioluminescence could be accurately measured during the cell cycle, heparin was added to schizonts prior to them rupturing. The heparin inhibits invasion and was removed once the schizonts began rupturing and then added back 2 hours later to produce an invasion window of 2 hours. The remaining unruptured schizonts were lysed in 5% sorbitol. The synchronized parasites were harvested every 4 hours until the 32–34 hour time point. Cultures were then diluted to 0.1% hematocrit and added to 1 volume of Nano-Glo Luciferase Assay Reagent and Nluc activity measured. Nluc values are represented as % of the maximum activity. Hours post invasion values are the mid-point of the 2 hour window. (TIF) [file pone.0112571.s005.tif]
